# Structural categorization and identification of electrostatic interactions in two proposed human serum albumin dimerization patterns and dipyridamole interaction

**DOI:** 10.55730/1300-0527.3769

**Published:** 2025-08-26

**Authors:** Haluk ÇETİNOK, Veyis KARAKOÇ, Erol ERÇAĞ, Yusuf Melih ŞEKERER, Hasan DEMİRCİ

**Affiliations:** 1 Department of Molecular Biology and Genetics, Faculty of Science, Koç University, İstanbul, Turkiye; 2 Department of Chemistry, Faculty of Science, Çankırı Karatekin University, Çankırı, Turkiye; 3 Department of Chemistry, Faculty of Engineering, Istanbul University-Cerrahpaşa, İstanbul, Turkiye; 4 Department of Pharmaceutical Chemistry, Faculty of Pharmacy, Biruni University, İstanbul, Turkiye; 5 Department of Molecular Biology and Genetics, Faculty of Science, Koç University, İstanbul, Turkiye; 6 Stanford PULSE Institute, SLAC National Laboratory, Menlo Park, CA, USA

**Keywords:** Human serum albumin, dipyridamole, molecular docking, protein-to-protein interactions, electrostatic interactions

## Abstract

Human serum albumin (HSA) is a ubiquitous, multifunctional protein responsible for the systemic distribution of both endogenous metabolites and exogenous pharmaceuticals. Its inherent properties, particularly its ability to seep into tissues and its multiple ligand-binding sites, have rendered HSA an attractive vehicle for nanoparticle-based drug delivery systems, particularly for cancer targeting. In this study, we present high-resolution crystallographic data revealing two distinct dimerization patterns of HSA (Protein Data Bank [PDB] ID: 9V61) obtained under high-concentration crystallization conditions, along with results from dipyridamole docking. Both dimer types demonstrate extensive interface areas and a significant number of electrostatic interactions. Comparative analysis with a previously reported dimer structure (PDB ID: 3JQZ) and other high-interface-area structures (PDB ID: 5Z0B, PDB ID: 8CKS) indicates similarities in contact regions but unique residue-level differences in bonding interactions. Interface surface area distribution and space group histograms further support the rarity and potential physiological relevance of the identified dimer forms. Importantly, these dimer configurations do not disrupt Sudlow’s drug-binding sites, as the dipyridamole docking analysis shows strong affinity for sites I and III without affecting their utility in engineered drug delivery. Our findings open new avenues for structure-based mutagenesis and nanoparticle design strategies centered on HSA dimerization dynamics.

## 1. Introduction

Human serum albumin (HSA) is a widely distributed globular serum protein produced in the liver that plays a crucial role in transporting metabolites and drug active ingredients throughout the human body [1]. Even though the name may suggest it predominantly localizes intravascularly, HSA is most abundant in the extravascular space, distributed in tissues and secretions [2]. HSA , with a half-life of 19 days, undergoes approximately 28 cycles of dislocating into tissues and returning to the vascular system via lymphatic vessels [1,2]. This distribution is mainly attributed to the caveolin-dependent albondin (gp60)-mediated transcytosis, which actively translocates albumin from the capillary lumen to the tissue lumen, transporting the nutrients albumin carries into the tissues [3]. In addition to the nutrients, HSA also carries a significant number of exogenous components, including the majority of prescribed drugs [4].

**Figure 1:**
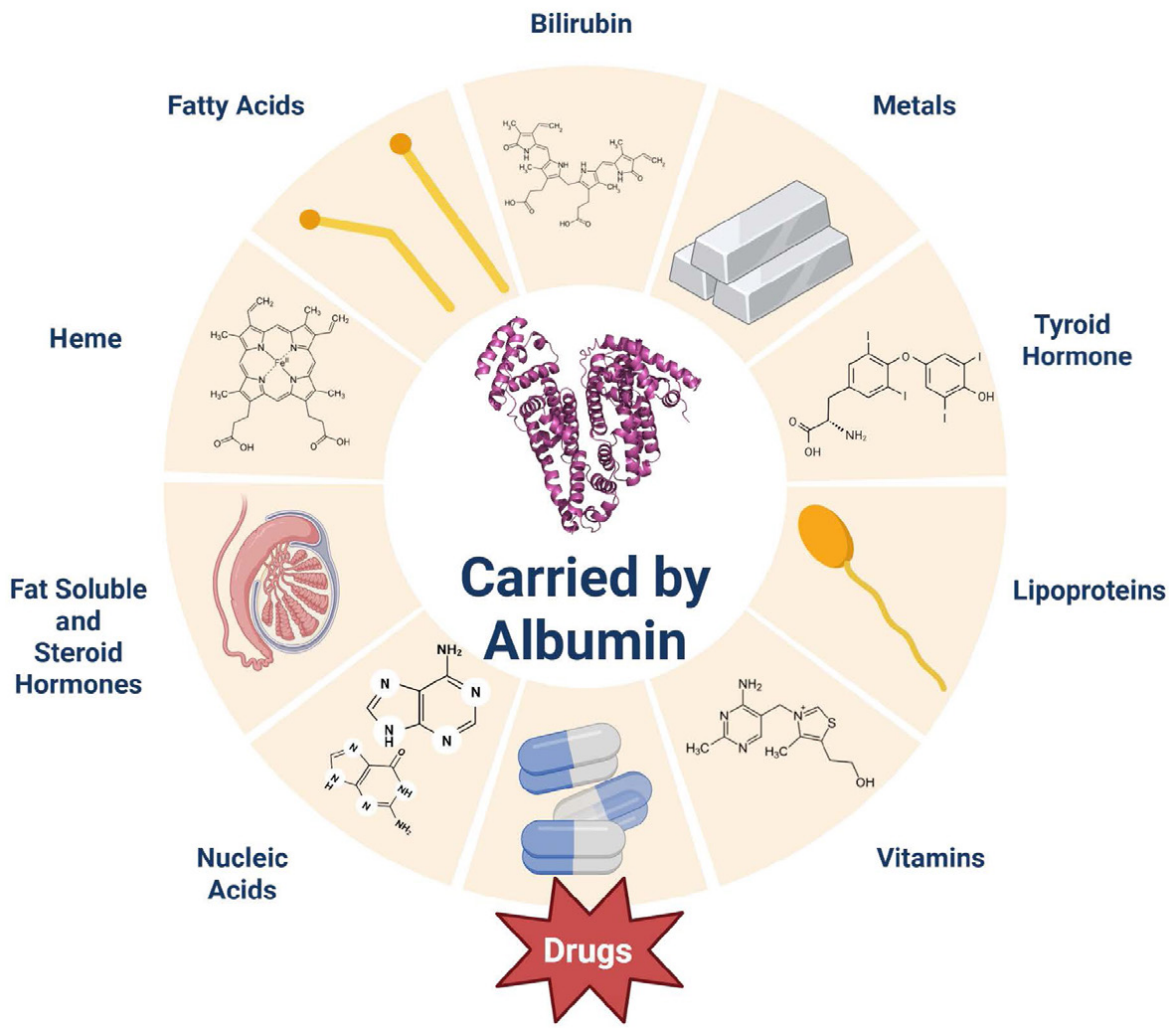
The scheme of some of the metabolites carried by or interacting with HSA. Starting at the top clockwise: Bilirubin, metals, etc.

Structurally, HSA exists as a collection of α-helices (67% α-helices, 10% turns, 23% random coils) that have two primary drug-binding sites, seven fatty acid-binding sites, and two nucleic acid-binding sites. [5,6]. Figure 1 shows a scheme summarizing the versatility of HSA, highlighting many of its ligands. HSA binding is a dynamic process consisting of the equilibrium between the association and dissociation of ligands; thus, it is dictated by the ligand concentration and free HSA amounts in a specific tissue, for example, as HSA carries the toxic bilirubin to the liver, only 20% of the bilirubin bound to HSA is loaded of in the hepatic capillaries demonstrating how albumin transportation occurs^1^

As HSA is an important and abundant nutrient carrier, multiple types of cancer overexpress gp60 receptors to better siphon those nutrients, rendering HSA an inherently cancer-targeting molecule [7,8]. This disproportionate targeting has led to an entirely different consideration: HSA nanoparticles [9]. HSA nanoparticles (either entirely made of HSA or covered with HSA) have at least a two-fold accumulation in tumor tissues as opposed to similar-sized nanoparticles without HSA [10]. Those nanoparticles can be prepared via either crosslinking or partially denaturing and renaturing HSA at high concentrations; hence, HSA self-interactions and multimerization can play an important role in the properties of those nanoparticles [9,10]. HSA consists of three homologous domains, each containing a double-repeating segment held together by 17 disulfide bridges. The helices are named according to their positions relative to the domain repeats.

**Figure 2:**
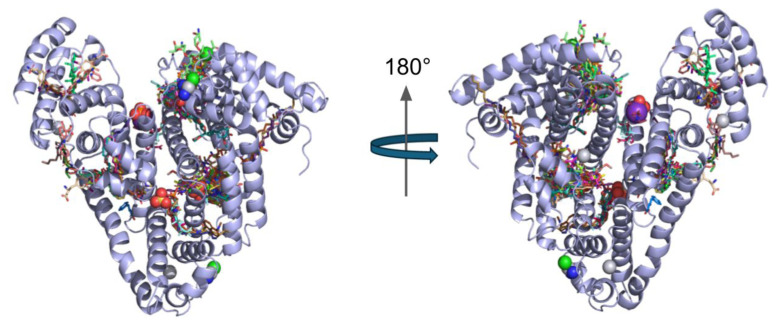
Most known ligands of human serum albumin are demonstrated in PDB deposited structures (over 100) aligned to our structure’s Chain A, emphasizing the versatility and the spread of binding sites on the protein.

Both within those repeats and between them, there are multiple binding sites for metabolites and drugs [2]. The metabolites and drugs with deposited crystal structures in the Protein Data Bank (PDB) have been aligned with our solved structure, Chain A, to emphasize the wide distribution of those binding sites across the protein in Figure 2. Fatty acids, drugs, metabolites (such as heme and bilirubin), and metals can bind to multiple sites on the protein, making every part of its surface relevant for drug transport and nanoparticle formation.

HSA can also interact with other proteins [11], and in blood, it is present at 5% as a covalent dimer with a disulfide bridge between two HSA monomers [12,13]. Even though there is little evidence of HSA forming reversible dimers in physiologic conditions [12,13], recombinantly dimerized HAS and HSA multimerized in nanoparticles have higher drug targeting efficiency [10,14]; hence, artificial and/or in vivo dimerization patterns may be utilized to improve nanoparticle efficiency and play a role in mutagenesis targeting. In crystallization, the dimers that form can either have biological significance or be a crystallization artefact; usually, the area of the interface between the monomers of the dimer and the interaction strength differentiate between them [15]. Non biological dimer interfaces tend to be small (<1000 Å) and involve little electrostatic interaction. The only other established HSA reversible dimer (PDB accession code: 3JQZ) has a similar surface area to our interfaces (1718.9 Å to 1695.1 Å) and comparable electrostatic interactions (28 to 24) [16]. While hydrophobic interactions are usually the driving force in protein dimers, electrostatic interactions, especially at higher concentrations, are not unheard of [17]. As HSA dimerizations and self-interactions can influence both nanoparticle dynamics and targeting properties, identifying possible dimerization patterns could introduce new parameters to both fields as mutagenesis targets and may offer insights into conditions under which HSA nanoparticles could be generated.

HSA contains several binding regions (Figure 1) with distinct properties, making this protein a significant factor in drug distribution in the bloodstream, as many pharmaceutical drugs bind to specific regions of the protein. For instance, drugs such as warfarin or phenylbutazone have a higher affinity toward Sudlow Site I. On the other hand, diazepam, ibuprofen, or naproxen demonstrate a higher affinity toward Sudlow Site II [18,19]. In the present study, molecular docking simulations of dipyridamole have been performed at Sudlow Site I of the IIA subdomain of HSA. Unfortunately, molecular docking has some limitations. One challenge is that the accelerated, simplified approach to protein and ligand flexibility assumes the protein is rigid, ignoring its dynamicity in endogenic conditions. This often fails to account for the rearrangements that occur during the induced fit between the protein and the ligand, leading to inaccurate binding poses. Additionally, the scoring functions used to estimate binding affinity rely on simplified energy terms and may omit solvent entropic contributions, resulting in poor correlation with experimental binding data. [20]

The active ingredient in the drug dipyridamole is mainly prescribed as an antiplatelet agent, as it inhibits phosphodiesterases, leading to the accumulation of signaling molecules cAMP and cGMP and, hence, inhibiting platelet activity^2^. Even though dipyridamole is not prescribed as an antiplatelet agent, it also has other effects, including increasing unassisted patency of synthetic arteriovenous hemodialysis grafts [21], inhibiting mengovirus DNA replication [22], and increasing cytotoxic activity against cancer cells compared with normal cells [23], making it a drug repurposing candidate. For a drug’s behavior within the patient, half-life, and distribution, and the aforementioned affinities, mainly HSA’s tendency to accumulate in cancer tissues, the drug interactions with the serum proteins can be crucial in manipulating said properties as well as developing novel therapeutic delivery methods regarding those drugs [24]. While there is little research on dipyridamole’s interaction with the HSA, experimental studies conducted spectrophotometrically show dipyridamole’s binding to Bovine Serum Albumin (BSA), raising the question of whether and how this binding translates to humans [25].

In this research, the initial aim was to observe the real-space binding of the HSA protein with the drug dipyridamole. However, in the experimentation, binding was not observed; instead, a potential homodimerization pattern for HSA was discovered and discussed. Circling back to dipyridamole dockings, these were compared with other established bindings.

## 2. Materials and methods

The HSA protein was purchased commercially and purified by gel filtration. The purified protein was then incubated with the drug dipyridamole and put through microdrop crystallization in terasaki plates. The acquired crystals were further analyzed by X-ray diffraction using our home-source Turkish DeLight and the software CrysAlisPro and Phenix. Once it became clear that the drug dipyridamole was not a part of the crystal docking, analyses were conducted.

### 2.1.1. Purification of HSA

The HSA used was the commercially available, intravenously administered medicine, Albuman (Centurion, 200 mg/mL). The protein was then filtered via gel filtration using Superdex 200 size-exclusion chromatography as a buffer-exchange step, with a buffer containing 150 mM NaCl and 20 mM Tris-HCl, pH 7.35.

### 2.1.2. Preparation of crystallization mixture

After purification, Amicon concentrators were used to concentrate the pure protein to 99 mg/mL. This final concentration was confirmed using a nanodrop spectrophotometer. The protein was then added to dipyridamole until its solubility limit (when undissolved drug precipitate was visible in an Eppendorf tube), and stored at 4 °C.

### 2.2. Crystallization of HSA

The protein sample was mixed with over 3000 crystallization solutions from the Kuybiigst-M (Koç University Structural Biology, Innovative Drug Design and Health Technologies Center) library in equal volumes (0.83 μL to 0.83 μL) and covered with 16.6 μL of paraffin oil, as described by Ertem et al. (2022) [26]. The protein was crystallized in the commercial crystallization solution Crystal Structure Screen (Hampton Research) #39, consisting of 2.0 M ammonium phosphate monobasic in 0.1 M Tris-HCl, pH 8.5. The crystals were first observed after three weeks of room-temperature incubation in the terasaki plate in the dark.

### 2.3. Data collection

Ambient temperature X-ray crystallographic data w ere collected using Rigaku’s XtaLAB Synergy R Flow XRD system according to the Turkish DeLight X-ray source protocols [27]. Multiple crystals were screened using the modified adapter of the XtalCheck-S plate reader. During data collection, due to protein precipitation, the crystals were not yet visible; upon searching in the well, multiple areas yielded X-ray diffraction data. The duration of exposure time was optimized to minimize the potential radiation damage caused by X-rays. Diffraction data were collected over several days, totaling approximately 10 h. The detector distance was set to 100.0 mm, and the scan width and plate angle were set to cover approximately 80°.

### 2.4. Data processing

The diffraction data were processed in CrysAlisPro [28,29] to complete automated data reduction. The collected data were then merged using the profit merge process with CrysAlisPro 1.171.42.59a software to produce an integrated reflection dataset (*.mtz) file for further analysis [27].

The *PHASER* implemented in the *PHENIX* suite was used for molecular replacement and refinement to fully position the folded protein chains into the three-dimensional electron map of the .mtz file [29]. The resulting .pdb protein structure file was then manually refined in COOT version 0.9 [30] and visualized in PyMOL software^3^. All processes up to this point were adapted from Atalay et al. (2023) [31].

### 2.5. Proteins, interfaces, structures, and assemblies (PISA) analyses

**Figure 3:**
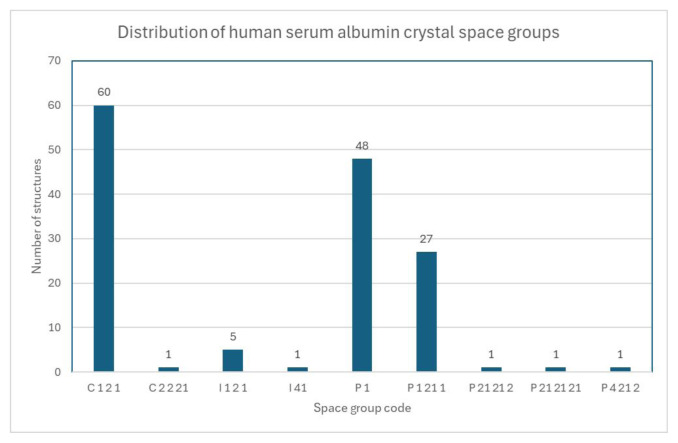
The histogram depicting the distribution of space groups for human serum albumin crystals across all crystal structures in the PDB, as well as heterogeneous (with more than one unique peptide chain) and noncrystal (cryo-EM and similar) structures, is excluded. The two proposed dimers in this paper (A–B and C–C) are included and counted in the I 1 2 1 space group.

**Figure 4:**
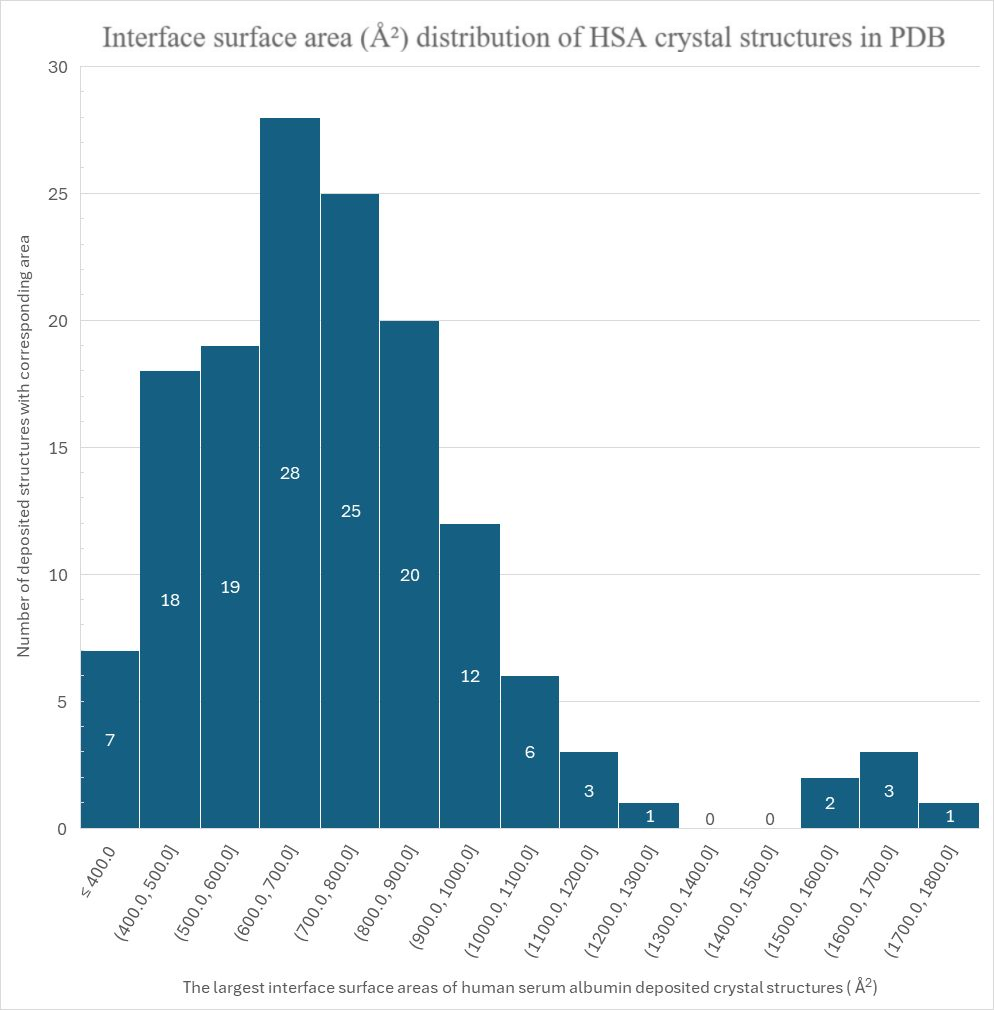
The histogram depicting the distribution of interface surface areas between human serum albumin chains in all HSA crystal structures in the PDB is heterogeneous (with more than one unique peptide chain) and noncrystal (cryo-EM and similar), and is therefore excluded. The two proposed dimers in this paper (A–B and C–C) are included and counted in the 1600.0 –1700.0 Å^2^ range.

The bond lengths, counts, and interface surface areas were calculated and generated using PDBePISA online software [32]. All deposited PDB unique HSA structures (excluding non crystal structures and those with non-HSA peptides) were processed by the software to generate the statistics presented in Figures 3 and 4.

### 2.6. Figures and tables

The figures were created using BioRender^4^ The software and the tables were created using Microsoft Excel. The f igures depicting protein foldings were prepared using PyMOL .

### 2.7. Molecular docking

Molecular docking calculations were performed using Autodock Vina software by Scripps Research Institute [33]. Three crystal structures of HSA with ligands bound to different binding sites have been downloaded from PDB . For Sudlow I, II, and III binding sites, 1H9Z (HSA protein complexed with warfarin) [34], 2BXH (HSA protein complexed with indoxyl sulfate) [18], and 4L8U (HSA protein complexed with 9-amino-camptothecin) had been downloaded, respectively. All of the crystal structures have resolutions higher than 2.50 Å.

The downloaded proteins were prepared using Molecular Graphics Laboratory (MGL) tools. First, the water molecules in the crystal structure were removed, and second, the amino acid residues with missing hydrogen atoms were protonated. Finally, the Kollman charges were added for the proteins. Following protein preparation, the ligands in the crystal structures were docked into the binding sites to validate the docking process by calculating root-mean-square deviations (RMSDs) between the positions of identical atoms in the crystalized ligands and the docked ligands.

Finally, the dipyridamole molecule was modeled and minimized using the MMFF94s force field in Avogadro [35] and prepared for AutoDock. After ligand preparation, dipyridamole was docked into the previously designated active site. Discovery Studio Visualizer [36] software was used to analyze the docking results.

## 3. Results

The solved structure’s data statistics were analyzed, leading to the structure discussed in this paper (PDB: 9V61). The data tables for this structure’s steps are shown in Table 1.

During data processing, the resolution cutoff was set to 2.50 Å because statistics from higher shells were lower than those used in the structure. The data collection and refinement statistics are presented in Table 1.

Other than a trimer [37] and another dimer (PDB accession codes: 5Z0B and 8CKS), there is no other crystal structure among the hundreds of structures in PDB with an interaction surface larger than 1300 Å, different from our two proposed dimers. In addition to the interaction surface area, our structures (PDB ID: 9V61) also have unusual space groups in their crystals. Our solved structures and 8CKS are *I 1 2 1*, and the only reversible dimer, 3JQZ, is the only *I 41*. Interface areas above 1300 Å2 are rare, and the specific space groups are shown in Figures 3 and 4, respectively.

**Figure 5:**
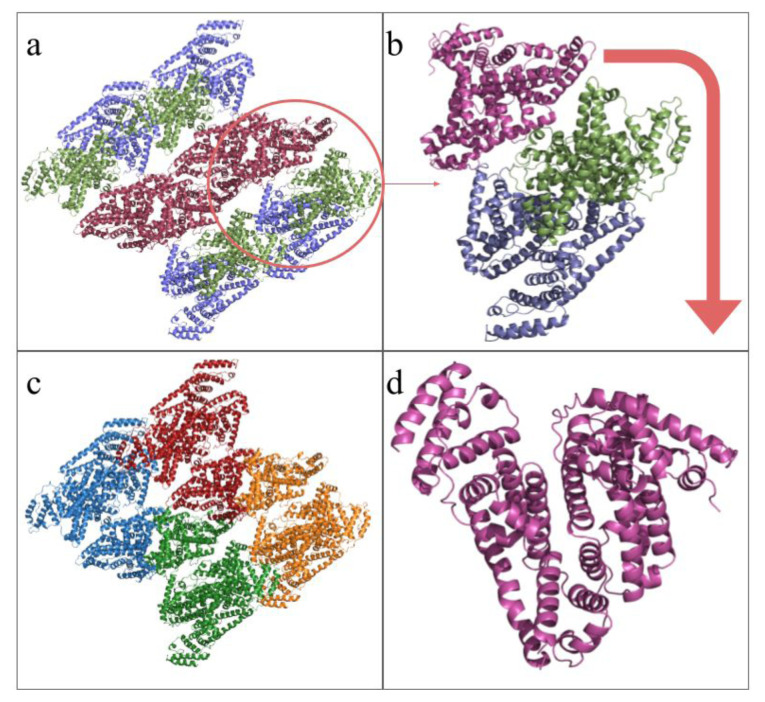
The components of the repeating and asymmetric units are colored and shown in a flowchart. (a) The repeating unit of the solved structures consists of four asymmetric units, each containing three HSA proteins, c olored by chains in the asymmetric unit. (b) A Single asymmetric unit consisting of three individual protein chains is extracted from the repeating unit. (c) The repeating unit with individual asymmetric units is colored differently. (d) Single HSA protein (Chain C) extracted from the asymmetric unit.

**Figure 6:**
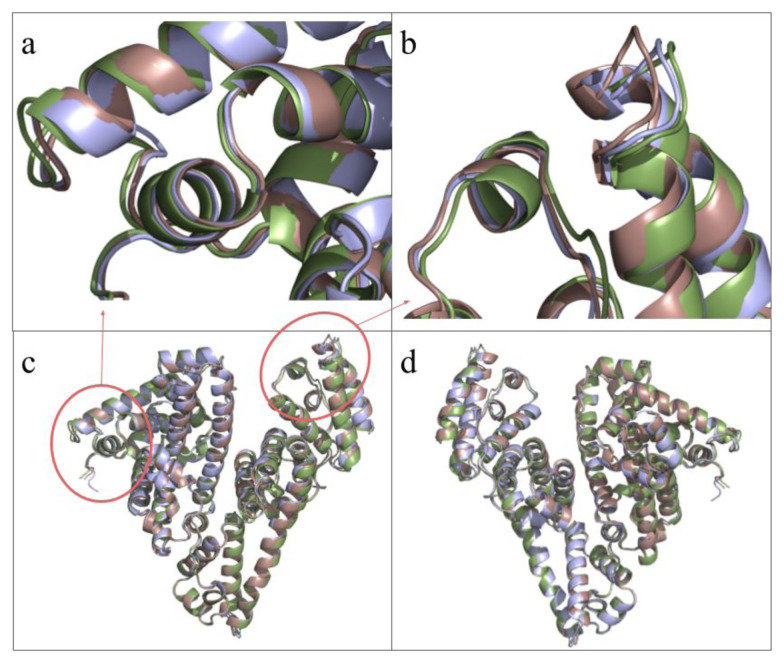
Three chains of our molecule are aligned at residues 365 –398. Chain B is colored green, and Chain C is colored brown. (a) This panel focuses on the slight shift between the helices of Ia-H3 and Ia-H4. (b) This panel focuses on the slight shift between the helices of IIIb-H3 and IIIb-H4. (c) This panel shows the overall alignment from the front. (d) Overall alignment from the back.

The histogram of the interface areas shown in Figure 4 has an empty range between 1300 Å^2^ and 1500 Å^2^, suggesting two separate normal distributions. It can be understood from Figure 5 that the space-filling of the repeating unit has two distinct dimerization patterns, the first being between Chains A and B, colored blue and green in Figure 5b, and the other being where two asymmetric units interlock into each other between their C hain C s. The alignment of the structure’s chains is shown in Figure 6.

The solved structure (PDB ID: 9V61) is presented in Figure 5. The assembly of the asymmetric unit into the repeating unit is presented as a flowchart; the entire repeating unit filling the I 1 2 1 space is shown in Figure 5a, with all unique protein chains colored individually. The single asymmetric unit is extracted into Figure 5 b. The repeating unit consists of four asymmetric units, as shown in Figure 5 c, each composed of three protein chains colored differently. A single HSA chain is shown in Figure 5 d.

Figures 6a and 6b focus on slight differences between the unique chains in the asymmetric unit, while Figures 6c and 6d provide front and back views of the overall alignment structure. RMSD values are A–B 0.461, A–C 0.522, and B–C 0.625.

**Figure 7:**
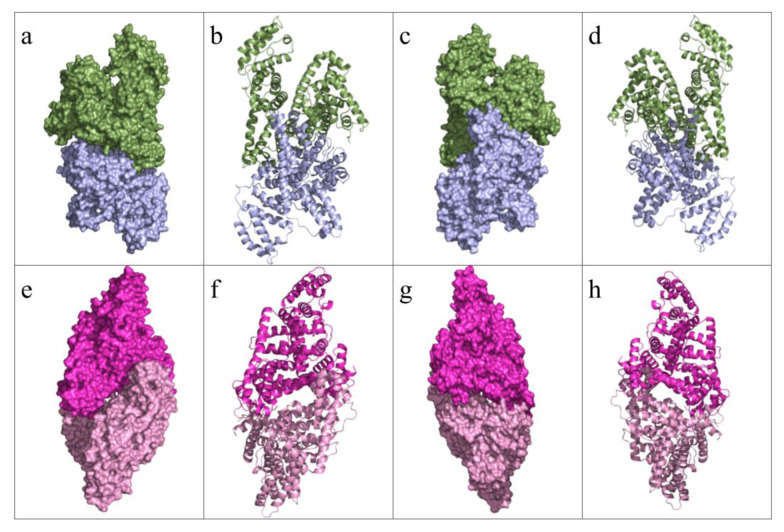
The two dimerized structures were solved. Chain A is colorized blue, Chain B is colorized green, Chain C is colorized light pink, and the other asymmetric unit’s Chain C is colorized in magenta. (a) The dimer of the Chains A and B, shown in the front view as surfaces. (b) The dimers of Chains A and B are shown in front view as cartoons. (a) The dimer of the Chains A and B, shown in the back view as surfaces. (b) The dimers of Chains A and B are shown in a back view as cartoons. (e) The dimer of Chains C1 and C2, shown in the front view as surfaces. (f) The dimer of the Chains C1 and C2, shown in the front view as cartoons. (g) The dimer of Chains C1 and C2, shown in the back view as surfaces. (h) The dimer of Chains C1 and C2, shown in the back view as cartoons.

The overall structures of these dimers are presented in Figure 7 as both surface and cartoon representations.

**Figure 8:**
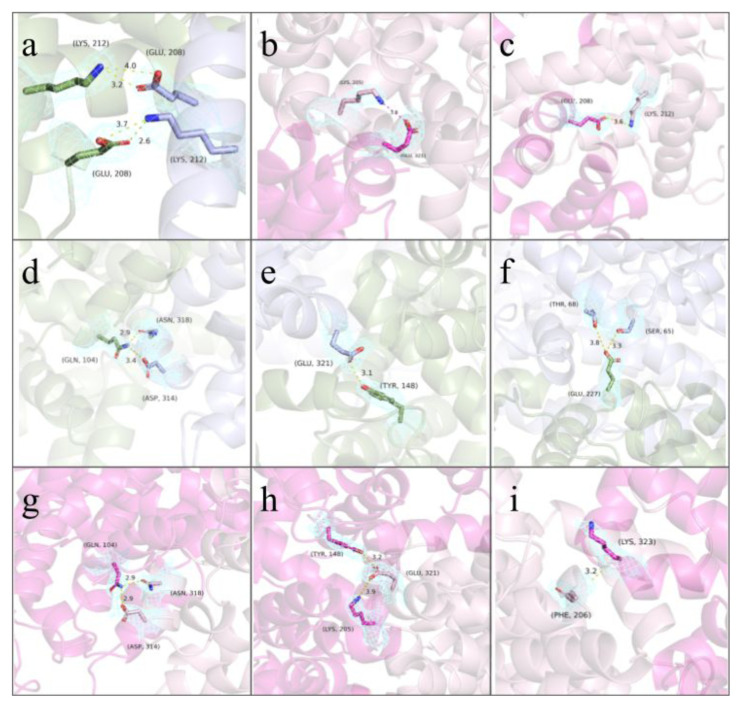
The PyMOL visuals demonstrate the bonds with the generated electron clouds. (a) The salt bridges presented by PISA between Chains A and B are shown with labels and distances. The cyan mesh corresponds to the electron cloud, the green-backboned protein is Chain B, and the blue-colored protein is Chain A. (b) The salt bridge numbered 2 (Chain C1 Lys205NZ and Chain C2 Glu321OE2) is shown. (c) The salt bridge numbered 3 (Chain C1 Lys212NZ and Chain C2 Glu208OE1) is shown. (d) The hydrogen bonds numbered 4 (Chain B Gln104NE2 and Chain A Asp314OD2) and 5 (Chain B Gln104NE2 and Chain A Asn318OD1) are shown. (e) The hydrogen bond numbered 6 (Chain B Tyr148OH and Chain A Glu321OE1) is shown. (f) The hydrogen bonds numbered 8 (Chain B Glu227OE2 and Chain A Ser65OG) and 9 (Chain B Glu227OE2 and Chain A Thr68OG1) are shown. (g) The hydrogen bonds numbered 14 (Chain C1 Asp314OD2 and Chain C2 Gln104NE2) and 15 (Chain C1 Asn218OD1 and Chain C2 Gln104NE2) are shown. (h) The hydrogen bonds numbered 16 (Chain C1 Glu321OE1 and Chain C2 Tyr148OH) and 17 (Chain C1 Glu321OE2 and Chain C2 Lys205NZ) are shown. (i) The hydrogen bond numbered 18 (Chain C1 Gln204O and Chain C2 Lys323N) is shown.

**Figure 9:**
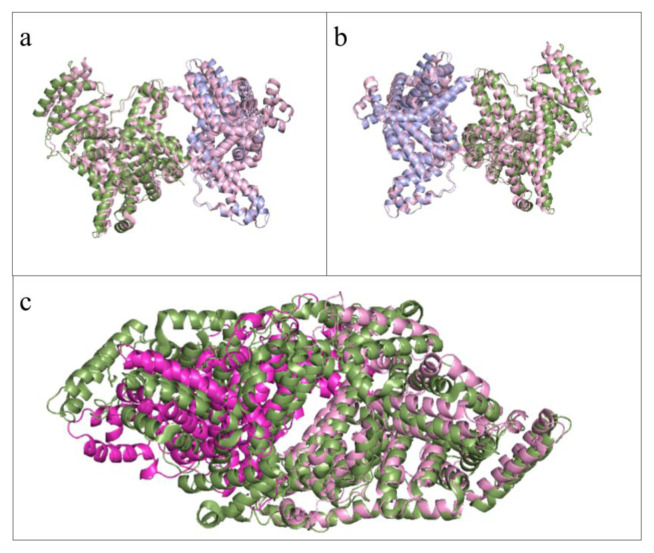
Our structure aligned to the other similar interface area structures 5Z0B (RMSD 1.0013) and 8CKS (RMSD 2.976), (a) the solved structure’s A (blue) and B (green) chains aligned to 5Z0B (pink) dimer, front view. (b) The solved structure’s Chains A (blue) and B (green) aligned with the 5Z0B dimer, viewed from the back. (c) The solved structure’s Chains C1 (pink) and C2 (magenta), aligned with the 8CKS (green) dimer, in a front view.

The two distinct dimerization patterns, A–B and C1–C2, were aligned with an RMSD value of 0.716. The salt-bridge contributors for both the A–B and C1–C2 dimers are presented in Table 2, and the hydrogen bonds between A–B are listed in Table 3. The C–C dimer hydrogen bonds are listed in Table 4. Some of those interactions are mapped onto their electron clouds (at an RMSD of 1.0) and shown in Figure 8. The solved structures (PDB ID: 9V61) proposed dimers’ alignment to the mentioned similar structures are presented in Figure 9, with RMSD values being 3JQZ: 3.784, 5Z0B: 1.0013, and 8CKS: 2.976.

### 3.1. Molecular docking

The crystallized ligands warfarin, indoxyl sulfate, and 9-amino-camptothecin were docked into their corresponding active sites. After the docking procedure, the positions of identical atoms between the crystallized ligand and the docked ligand were compared, and the parameter of RMSD was calculated. RMSD calculation is a crucial method for validating the docking procedure [38], and an RMSD value below 2 Å indicates that the docking procedure is close to the experimental result. Using the DockRMSD script [39], the RMSD value of our docked ligand was calculated. The calculated RMSD values are given in Table 5.

After the validation of the docking procedure, dipyridamole is docked into different binding sites of HSA. The binding energies of dipyridamole to Sudlow I, II, and III sites are given in Table 6.

Molecular docking results show that dipyridamole demonstrates significant affinity toward Sudlow Sites I and III of the HSA protein by forming several intermolecular interactions with the residues of the active sites. Furthermore, it has been shown that dipyridamole has no affinity toward Sudlow Site II due to high intermolecular repulsions and the small size of the binding site.

#### 3.1.1. Sudlow Site I

**Figure 10:**
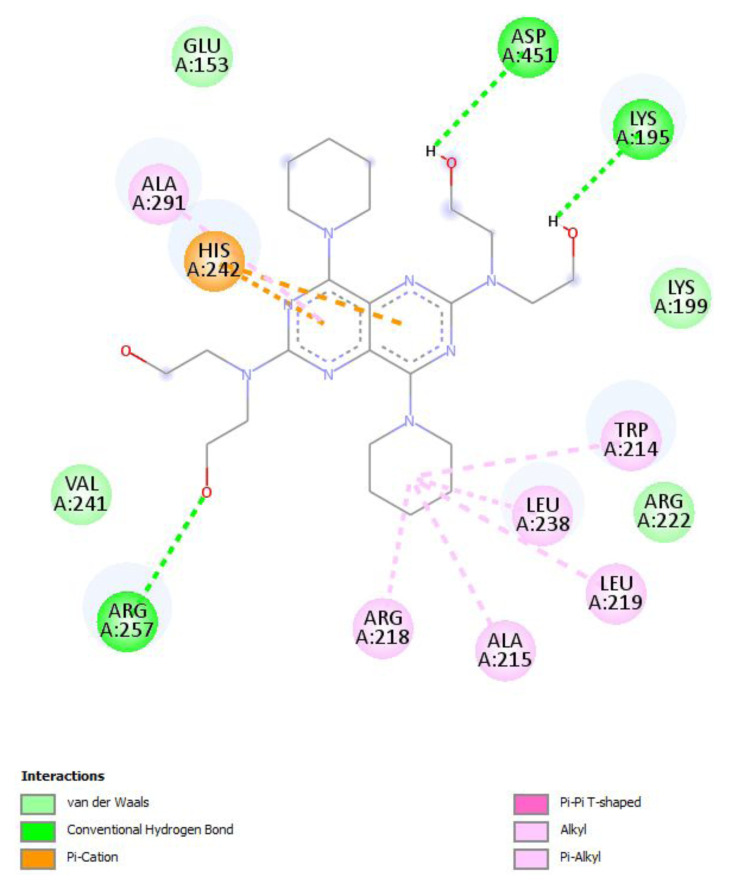
The 2D interaction map between dipyridamole and the amino acid residues of Sudlow Site I is depicted.

**Figure 11:**
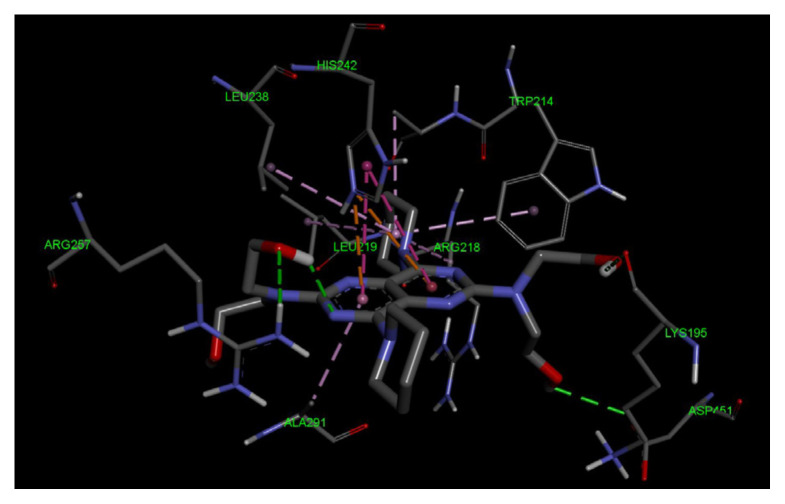
The 3D interaction map between dipyridamole and the amino acid residues of Sudlow Site I is depicted.

Molecular docking of dipyridamole toward Sudlow Site I in the IIA subdomain of HSA protein has shown that dipyridamole has demonstrated significant binding affinity to Sudlow Site I, as shown in Figure 10 and as a three-dimensional (3D) image in Figure 11. The two-dimensional (2D) map of dipyridamole and Sudlow Site I shows that dipyridamole’s aromatic rings form strong π-π interactions with the HIS242 residue and π-alkyl interactions between TRP 214, ALA215, LEU219, LEU238, and ALA291 residues. Furthermore, dipyridamole forms strong hydrogen bonds with LYS195, ARG257, and ASP451 residues with its hydroxyl tails at the two ends of dipyridamole.

#### 3.1.2. Sudlow Site III

**Figure 12:**
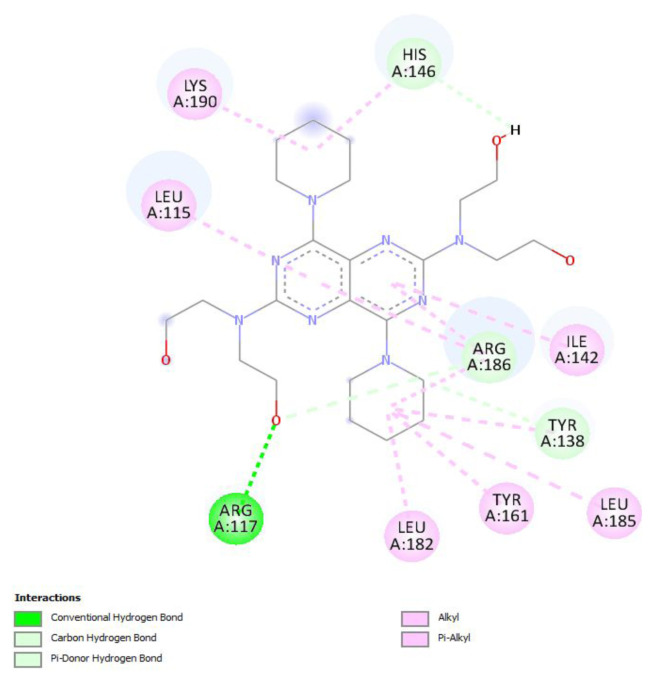
The 2D interaction map between dipyridamole and the amino acid residues of Sudlow Site III is depicted.

**Figure 13:**
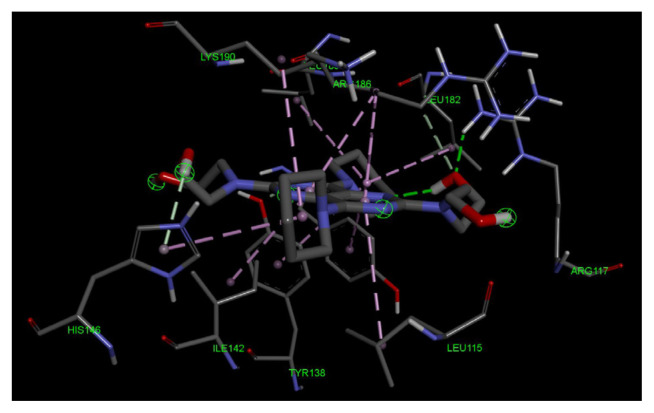
The 2D interaction map between dipyridamole and the amino acid residues of Sudlow Site III is depicted.

Molecular docking of dipyridamole toward Sudlow Site III in the IB domain of HSA protein shows that dipyridamole demonstrates significant affinity toward Sudlow Site III, as shown in Figure 12 and as 3D in Figure 13. As depicted in the 2D interaction map, dipyridamole forms a strong hydrogen bond between its hydroxy tail and the ARG117 residue of the active site. Furthermore, dipyridamole forms several π interactions between its aromatic ring and heteroatomic saturated ring and the LEU115, HIS146, TYR161, ILE142, LEU182, LEU185, LEU190 residues of the active site.

## 4. Discussion

HSA dimerization patterns can offer novel insights into nanoparticle generation and recombinant-HSA-dependent delivery systems; thus, this research identifies two distinct dimerization patterns. Protein dimerization can be facilitated by electrostatic interactions, such as salt bridges and hydrogen bonds; therefore, the proposed HSA dimerization is characterized and identified as shown in Table 2, Table 3, and Table 4 [40].

The interface area histogram in Figure 4 shows that the distribution of interface area in HSA crystals is bimodal, with the first peak corresponding to monomer crystallization and the second, smaller peak suggesting that dimerization occurs before nucleation of crystallization. The two peaks have a large gap between them, indicating that the events leading to crystallization are different. The concentration of unusual space groups, such as I 41 and I 1 2 1, is also a notable difference, further suggesting that the crystallized units are different.

The overwhelming interface area of the 9V61 structure and a comparatively large number of electrostatic interactions suggest [41] an inherent interaction in the dimers that is different from crystallization artefacts, suggesting somewhere in the process, possibly within the crystallization droplet, HSA monomer –dimer equilibrium shifted to favor dimers more. When compared with the other structures with unusually high interface areas in Figure 9, it is observed that differences exist between the structures, even though the interacting surfaces are the same, with slight differences in interacting residues. Figure 6 displays the differences among three distinct 9V61 chains, showing that the differences reflected in the RMSD values are not concentrated in small areas or in Sudlow’s sites, thereby emphasizing that the theorized drug-binding properties do not change within the chains. In Figure 8, electrostatic interactions were shown, with electron clouds visible to confirm that this protein folding is reliable and that the interacting residues are observed rather than calculated during molecular replacement. While the interfaces of A-B, C1-C2, 5Z0B, and 8CKS are similar, with multiple overlapping pairings, each also has unique pairings identified by PDBePISA analyses; hence, they are regarded as separate patterns, as these differences may be significant and warrant follow-up nanoparticle research.

In HSA-based drug delivery, it is important to tailor the approach to the drug and its binding site on the protein. Our dimerization patterns do not alter the Sudlow drug-binding sites and hence would not negatively affect the binding of most drug-active ingredients. It may also be possible to alter the strength, association, and dissociation patterns of HSA nanoparticles and recombinant-dimer-HSAs by mutating the identified residue via mutagenesis or targeted post translational modifications. The increasing knowledge of possible HSA dimerization patterns may introduce new parameters, the protein itself, to the area of HSA nanoparticles; hence, we recommend nanoparticle efficiency comparison research with HSA mutants, which may lead to important steps in the field.

While the initial gel filtration was at 7.35 pH, the 8.5 pH crystallization condition had five times the buffer salt, meaning the crystallization drop was close to 8.0 pH, which can cause deviations from physiological behavior. The dipyridamole binding may have inhibited this difference. Similarly, these divergences can also be attributed to the nonphysiological pattern within the crystal structure. The gel filtration showed a single monomer peak, indicating that the interface became significant within the drop during crystallization. While not biologically significant, this pattern can still be regarded as a possible aggregation or self-interaction surface during HSA-nanoparticle generation in an artificial environment.

Molecular docking results show that dipyridamole shows significant affinity toward Sudlow Site I and III binding regions, while showing poor affinity toward Sudlow Site II.

Dipyridamole’s low affinity toward Sudlow Site II might be due to the small size of Sudlow Site II, resulting in an unfavorable energy calculation in the docking process. Furthermore, it can be inferred that binding events may affect the distribution of dipyridamole in the bloodstream, thereby directly affecting its pharmacokinetic profile [42].

Another similar molecular docking study was conducted on bovine serum albumin (BSA) by Karakoç et al. [43]. In that study, because no crystal structures of BSA with ligands bound to its active site were available, they scanned potential binding pockets using their software and performed molecular docking to these regions. In comparison, since HSA is better studied, we were able to obtain crystal structures of HSA with ligands bound to different binding regions reported in the literature (Sudlow S ites I, II, and III). Combining the findings of both studies, given that the structures of BSA and HSA are highly similar, it can be concluded that dipyridamole may bind to HSA via other binding modes that are independent of the known drug-binding sites reported in the literature.

## 5. Conclusion

The identification and characterization of two novel HSA dimerization interfaces of 9V61 provide compelling evidence for previously unregarded plasticity in albumin self-interaction. The extensive electrostatic and hydrogen-bonding interactions, coupled with their large interfacial surface areas, suggest that these dimers may have biological or application-relevant significance rather than being mere crystallographic artefacts. As the interactions listed in Tables 2, 3, and 4 do not compromise known pharmacologically relevant binding domains, they present promising frameworks for engineering stable HSA-based nanoparticles. Moreover, these dimerization patterns propose a new variable in the field of albumin nanoparticle engineering—namely, the modulation of protein –protein interfaces. Future investigations into mutagenesis or post translational modifications targeting the residues involved in these dimer interfaces may yield tunable nanoparticle constructs with altered stability and targeting efficiency. These insights not only contribute to the structural biology of HSA but also reinforce the usage of HSA dimers in advanced drug delivery platforms. As it concerns the dipyridamole interaction with Sudlow Sites I and III, we have calculated comparative binding energies for previously established ligands, indicating that dipyridamole is expected to bind both Sudlow Sites I and III.

## Figures and Tables

**Table 1:** The X-ray crystal structure statistics.

PDB ID	9V61		
**Data collection**			
X-ray source	Turkish DeLight	No. reflections	61139
Temperature (K)	298	Rwork / Rfree	0.254/0.320
Space group	*I* 1 2 1	**No. atoms**	
a,b,c (A˚)	87.80, 116,49, 205,46	Protein	13599
α,β,γ (°)	89.9, 99.25, 90.02	Ligand / Ion / Water	348
Resolution	29.41–2.50	Coordinate errors	0.61
CC1/2	0.919	Bond lengths (˚A)	0.010
CC*	0.979	Bond angles (°)	1.332
I/σI	3.8	**Ramachandran plot**	
Completeness (%)	94.9	Favored (%)	95.2
**Refinement**		Allowed (%)	4.5
Resolution	2.5Å	Disallowed (%)	0.30

PDB ID	9V61		
**Data Collection**			
X-ray source	Turkish DeLight	No. reflections	61139
Temperature (K)	298	Rwork / Rfree	0.254/0.320
Space Group	I 1 2 1	**No. atoms**	
a,b,c (A˚)	87.80, 116,49, 205,46	Protein	13599
α,β,γ (°)	89.9, 99.25, 90.02	Ligand / Ion / Water	348
Resolution	29.41–2.50	Coordinate errors	0.61
CC1/2	0.919	Bond lengths (˚A)	0.010
CC*	0.979	Bond angles (°)	1.332
I/σI	3.8	**Ramachandran plot**	
Completeness (%)	94.9	Favored (%)	95.2
**Refinement**		Allowed (%)	4.5
Resolution	2.5Å	Disallowed (%)	0.30

**Table 2:** The PISA-proposed salt bridges between both A-B chains and the two C chains are listed, including the bonding atoms and bond distances.

	Chain B	Chain A	Distance (Å)		Chain C1	Chain C2	Distance (Å)
**1**	LYS 212 [NZ]	GLU 208 [OE1]	3.16	**1**	LYS 93 [NZ]	ASP 308 [OD1]	3.49
**2**	LYS 212 [NZ]	GLU 208 [OE2]	3.99	**2**	LYS 205 [NZ]	GLU 321 [OE2]	3.86
**3**	GLU 208 [OE1]	LYS 212 [NZ]	2.63	**3**	LYS 212 [NZ]	GLU 208 [OE1]	3.58
**4**	GLU 208 [OE2]	LYS 212 [NZ]	3.71	**4**	ASP 308 [OD1]	LYS 93 [NZ]	3.49
				**5**	GLU 321 [OE2]	LYS 205 [NZ]	3.86
				**6**	GLU 208 [OE1]	LYS 212 [NZ]	3.58

**Table 3:** The PISA-proposed hydrogen bonds between the A and B chains, with the bonding atoms and bond distances listed.

	Chain B	Chain A	Distance (Å)		Chain B	Chain A	Distance (Å)		Chain B	Chain A	Distance (Å)
***1***	LYS 323 [N]	GLN 204 [O]	3.22	**6**	TYR 148 [OH]	GLU 321 [OE1]	3.14	**11**	ASP 314 [OD2]	GLN 104 [NE2]	3.59
***2***	LYS 212 [NZ]	GLU 208 [OE1]	3.16	**7**	GLU 266 [O]	LYS 4 [NZ]	3.69	**12**	GLU 321 [OE1]	TYR 148 [OH]	3.61
***3***	SER 65 [OG]	GLU 227 [OE1]	3.10	**8**	GLU 227 [OE2]	SER 65 [OG]	3.34	**13**	ALA 320 [O]	LYS 205 [NZ]	2.70
***4***	GLN 104 [NE2]	ASP 314 [OD2]	3.45	**9**	GLU 227 [OE2]	THR 68 [OG1]	3.81	**14**	GLU 208 [OE1]	LYS 212 [NZ]	2.63
***5***	GLN 104 [NE2]	ASN 318 [OD1]	2.91	**10**	ASN 318 [OD1]	GLN 104 [NE2]	2.98	**15**	GLN 204 [O]	LYS 323 [N]	3.22

**Table 4:** The PISA-proposed hydrogen bonds between the two C chains are listed, along with the bonding atoms and bond distances.

	Chain C1	Chain C2	Distance (Å)		Chain C1	Chain C2	Distance (Å)		Chain C1	Chain C2	Distance (Å)
***1***	LYS 4 [NZ]	GLU 266 [O]	2.68	**7**	TYR 148 [OH]	GLU 321 [OE1]	3.20	**13**	GLU 227 [OE2]	THR 68 [OG1]	3.32
***2***	SER 65 [OG]	GLU 227 [OE1]	3.48	**8**	LYS 205 [NZ]	GLU 321 [OE2]	3.86	**14**	ASP 314 [OD2]	GLN 104 [NE2]	2.86
***3***	SER 65 [OG]	GLU 227 [OE2]	3.14	**9**	LYS 323 [N]	GLN 204 [O]	3.24	**15**	ASN 318 [OD1]	GLN 104 [NE2]	2.89
***4***	THR 68 [OG1]	GLU 227 [OE2]	3.32	**10**	GLU 266 [O]	LYS 4 [NZ]	2.68	**16**	GLU 321 [OE1]	TYR 148 [OH]	3.20
***5***	GLN 104[NE2]	ASN 318 [OD1]	2.89	**11**	GLU 227 [OE1]	SER 65 [OG]	3.48	**17**	GLU 321 [OE2]	LYS 205 [NZ]	3.86
***6***	GLN 104 [NE2]	ASP 314 [OD2]	2.86	**12**	GLU 227 [OE2]	SER 65 [OG]	3.14	**18**	GLN 204 [O]	LYS 323 [N]	3.24

**Table 5:** Docking results of crystallized ligands and RMSD values.

Crystallized ligand	PDB ID	Active site	Docking score (kcal/mol)	RMSD (Å)
Warfarin	1H9Z	Sudlow Site I (IIA)	− 9.1	0.706
Indoxyl sulfate	2BXH	Sudlow Site II (IIIA)	− 7.4	1.312
9-Amino-camptothecin	4L8U	Sudlow Site III (IB)	− 10.8	0.584

**Table 6:** Docking scores of dipyridamole in different active sites.

PDB ID	Active Site	Docking Score (kcal/mol)
1H9Z	Sudlow Site I (IIA)	− 7.4
2BXH	Sudlow Site II (IIIA)	+ 10.4
4L8U	Sudlow Site III (IB)	− 7.6

## Footnotes

1 NCBI (2022) Physiology, Bilirubin [online]. Website https://www.ncbi.nlm.nih.gov/books/NBK470290/ [accessed 15 June 2025].

2 CBI (2022) Dipyridamole [online]. Website https://www.ncbi.nlm.nih.gov/books/NBK554455/ [accessed 15 June 2025].

3 PyMOL [online]. website http://www.pymol.org/pymol. [Accessed 15 June 2025].

4 BioRender [online]. Website https://www.biorender.com/. [Accessed 15 March 2025].
